# Functional responses of white spruce to snowshoe hare herbivory at the treeline

**DOI:** 10.1371/journal.pone.0198453

**Published:** 2018-06-01

**Authors:** Justin Olnes, Knut Kielland, Hélène Genet, Glenn P. Juday, Roger W. Ruess

**Affiliations:** 1 Department of Biology and Wildlife, University of Alaska Fairbanks, Fairbanks, Alaska, United States of America; 2 Institute of Arctic Biology, University of Alaska Fairbanks, Fairbanks, Alaska, United States of America; 3 School of Natural Resources and Agricultural Sciences, University of Alaska Fairbanks, Fairbanks, AK, United States of America; Helmholtz Centre for Environmental Research - UFZ, GERMANY

## Abstract

Herbivores can modify the rate of shrub and treeline advance. Both direct and indirect effects of herbivory may simultaneously interact to affect the growth rates of plants at this ecotone. We investigated the effect of snowshoe hare herbivory on the height of white spruce at two treeline locations in Alaska, USA. White spruce is expanding its distribution both upwards in elevation and northward in latitude because of climate warming, and snowshoe hares are already present in areas likely to be colonized by spruce. We hypothesized that herbivory would result in browsed individuals having reduced height, suggesting herbivory is a direct, negative effect on spruce treeline advance. We found an interactive effect between browsing history and spruce age. When young (under 30 years old), individuals that were browsed tended to be taller than unbrowsed individuals. However, older seedlings (over 30 years old) that had been browsed were shorter than unbrowsed individuals of the same age. Hares suppress faster growing individuals that are initially taller by preferentially browsing them as they emerge above the winter snowpack. This reduced height, in combination with increased mortality associated with browsing, is predicted to slow the advance of both latitudinal and altitudinal treeline expansions and alter the structure of treeline forests.

## Introduction

Herbivory has direct and indirect effects on the rate of shrub expansion and treeline advance [1, 2]. Directly, herbivory may reduce growth rates via biomass consumption and meristem removal [3, 4] that may lead to plant mortality. By contrast, the presence of herbivores may benefit the growth of less palatable species by reducing competition from neighbors [5,6], but also benefit the growth of palatable species by inducing compensatory growth [7, 8]. Predicting the response of vegetation is complicated when direct (i.e. alteration of growth rate) and indirect (i.e. change in species competition) effects act simultaneously.

In Alaska, white spruce (*Picea glauca* (Moench) Voss) is gradually advancing upward in elevation and northward in latitude, replacing tundra landscapes with boreal forest [9, 10]. Preceding treeline advance is the expansion of tall deciduous shrubs, which provides suitable habitat for snowshoe hares (*Lepus americanus* L.) to occupy landscapes currently, or soon to be, colonized by white spruce [11, 12]. Snowshoe hares are dominant boreal herbivores that have the capacity to modify vegetation dynamics, including the establishment of white spruce via their browsing of seedlings during winter [13]. Whereas treeline spruce typically experience the negative consequences of direct herbivory by hares, they may also accrue benefits when hares browse the surrounding, more palatable vegetation. Thus, the functional responses of white spruce to herbivory, in terms of growth, seed production, and establishment, reflect the outcome of these negative and positive effects.

In light of previous studies demonstrating the capacity for hares to significantly reduce spruce height in lowland floodplains [4, 14], we investigated whether treeline spruce would also experience reduced height as a result of snowshoe hare herbivory. We addressed this question by measuring the height, age, and browsing history of white spruce seedlings at two treeline locations in Alaska: at the altitudinal limit of white spruce in Denali National Park, and at the latitudinal limit of the species near the Middle Fork of the Koyukuk River. We hypothesized that browsed individuals would have reduced height growth compared to individuals that had not been browsed, as indicated by changes in the slope of the relationship between spruce height and age. To further understand the role of associated deciduous vegetation in altering spruce height at treelines, we additionally hypothesized that the proportion of surrounding deciduous vegetation browsed would positively relate to spruce height, reflecting an indirect benefit of hare browsing.

## Methods

### Study locations and sampling design

**Fig 1:**
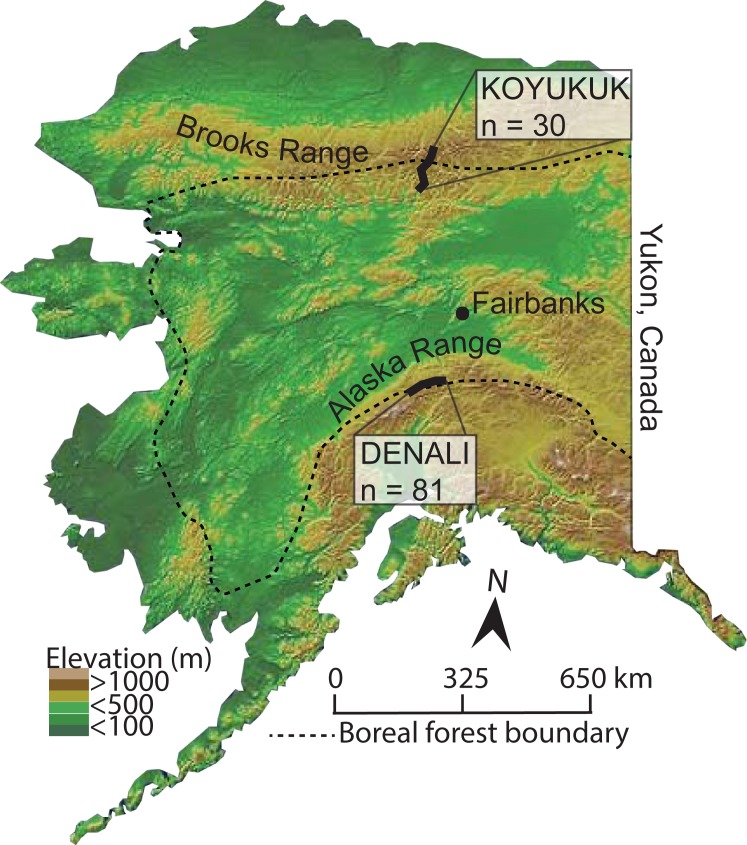
Locations of sampling regions, Denali and Koyukuk, in Alaska, USA.

Denali National Park and Preserve (Denali), and the Middle Fork of the Koyukuk River (Koyukuk) include extensive areas representative of the altitudinal and latitudinal limits of white spruce in Alaska, respectively (Fig 1). Snowshoe hares are present in both areas and periodically achieve very high abundances [15].

Denali (63°43’N, 148°57’W) is located within the Alaska Range, approximately 130 km southwest of Fairbanks. Lower elevations are dominated by white and black spruce (*P*. *mariana* (Mill.) B.S.P.) forests, which transition to alpine shrub tundra at higher elevations [16]. Nine sites were accessed along the 143-km park road corridor via hiking. For each site, we established a transect 1.5–5 km long, oriented perpendicular to the hillslope. Along these transects, we established 25 m^2^ plots at 75 m elevation intervals between 500–1175 m a.s.l. These plots encompassed the elevational range of white spruce as determined by Roland et al. [16]. Because floodplain and upland vegetation communities are distinct, and floodplains represent high-quality hare habitat and optimal conditions for spruce establishment [12], additional plots (n = 38 floodplain plots total) were positioned along major stream or river drainages near each elevational transect (500–945 m a.s.l.). Plot number varied between 4 and 12 plots per transect site (n = 81 plots total), based on the range of elevations present at each location and the accessibility of each plot. We specifically addressed variation in spruce establishment and snowshoe hare herbivory along elevational gradients and between habitat types in a separate study [12].

Located on the southern flanks of the Brooks Range, the Koyukuk study area (67°25’N, 150°07’W) is approximately 310 km north of Fairbanks. Sites were located between 365 and 790 m a.s.l. and were accessed from the Dalton Highway. Ten sites were selected along a latitudinal gradient in which the most northerly site represented the farthest north white spruce trees along the highway corridor. At each of the 10 sites, we sampled three 25 m^2^ plots (n = 30 plots total). Plot locations were chosen randomly using satellite images prior to field sampling and included both floodplain (n = 18 plots) and upland (n = 12 plots) locations. All plots were located below the local altitudinal tree limit.

In the summer of 2015, we sampled all individual white spruce within each 25 m^2^ plot at both Denali and Koyukuk, and measured the height and basal diameter, and recorded browsing history for each individual as either “no browsing”, “moderate browsing”, or “severe browsing”. Moderate browsing was defined as 1–2 apical browsing events, and severe browsing was defined as > 2 apical browsing events or more than half of all lateral branches being browsed. This is a conservative estimate of browsing history as older browse scars may no longer be apparent, however, we consider this hidden browse history rare for spruce seedlings and saplings as browse scars often remain visible for several decades [12,13]. Hare browse events were identified by the sharp 45° angle of the bite and we back-counted current annual growth segments to estimate the year when the apical meristem was browsed. Similarly, we determined the age of most individual spruce trees using whorl counts (the number of apical annual growth segments [13]). For trees with annual growth segments that were too obscured to accurately age in this manner, we collected basal core samples and determined tree age using standard techniques of tree ring counting [17]. To estimate the age of each tree when browsed, we subtracted the year of browsing from the year of establishment for each tree. Within each plot, we also used four 1 m^2^ subplots to estimate the density of deciduous woody vegetation (ramets m^-2^) and associated browsing by hares (browsed ramets m^-2^). We did not sample browsing of herbaceous vegetation because our study focused on winter browsing, when hares primarily eat woody vegetation [18, 19]. Individual deciduous ramets were defined as a single stem rooted in the ground within the 1 m^2^ subplot and counted as being browsed if any subsequent branches off this ramet had apical growth points removed by hares. We then calculated the proportion of deciduous woody ramets browsed from these values. Values were averaged across each subplot to obtain a single proportion of ramets browsed per plot for each species.

### Analysis

We performed all statistical analyses using the R statistical software version 3.4.3 [20]. Assumptions of equal variance and normality of the residuals were confirmed graphically for all linear models. Statistical significance (α) was set at 0.05.

We used a linear mixed effect model to test the effects of browse history on spruce height for all spruce < 200 cm tall (function: lmer), encompassing the height range for which spruce are vulnerable to browsing by hares [21]. Our response variable was spruce height, and our fixed effects were spruce age, browsing history and their interaction term, the proportion of associated deciduous vegetation browsed, and habitat type (floodplain or upland). We included the proportion of associated deciduous vegetation browsed to test our hypothesis that indirect browsing by hares may have a positive influence on spruce height. Although our primary goal was to assess the direct and indirect effects of hare browsing specifically, we included the habitat type term because we suspected that floodplain spruce would have significantly greater height than upland spruce for both Denali and Koyukuk as floodplain habitats tend to be more productive. Because our analysis was conducted at the level of individual tree, we avoided pseudoreplication by including sampling region (Denali or Koyukuk) and plot nested within region as random effects. We log-transformed the response variable to improve model fit and meet model assumptions for our full model. We then developed competing models that incrementally excluded our fixed effects and selected the best model based on Bayesian Information Criterion scores (BIC). BIC is a parsimonious method of model selection that accounts for model fit while penalizing for the number of parameters included in the model. We selected the model with the lowest BIC score as our final model for spruce height (function: bic), and only considered additional models if their BIC score differed by less than 2 from the lowest BIC score. We determined the significance of each fixed effect within our final model using F tests (function: anova) and compared the means among browsing categories using pairwise comparisons for mixed effects models (function: lsmeans). We determined whether model coefficients significantly differed from zero using t tests (function: lmer). We calculated both the marginal ($R_{m}^{2}$) and conditional ($R_{c}^{2}$) R-squared values for our model (function: r.squaredGLMM), which give the amount of variation explained by the fixed effects alone and by the fixed and the random effects, respectively [22]. To estimate the amount of variation in spruce height explained by browsing, we also calculated the marginal R-squared for our final model without hare browsing and compared this to the $R_{m}^{2}$ of the model that included the browsing by age interaction.

## Results

**Fig 2:**
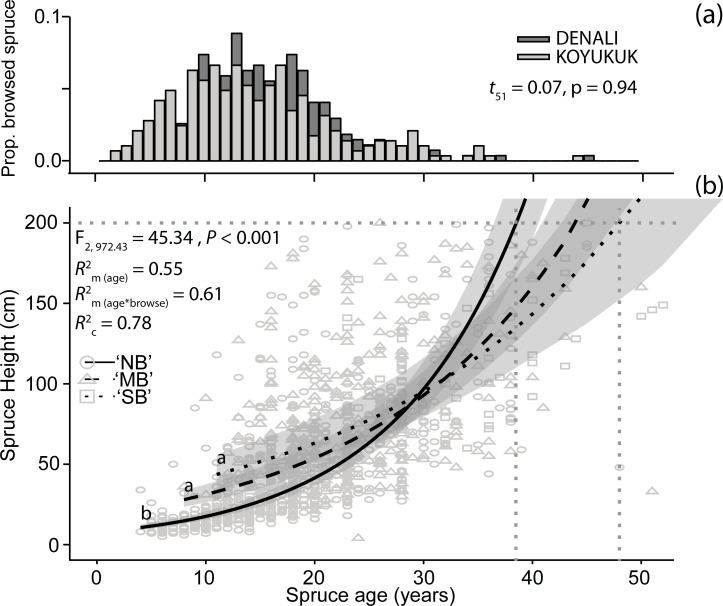
(a) Most spruce were 10–20 years of age when browsed at both Denali and Koyukuk. (b) Browsing history (no browsing (NB), moderate browse (MB), severe browse (SB)) alters the relationship between spruce height (cm) and age (years). Stippled gray lines indicate age difference of escaping herbivory (height = 200 cm) for browsed or not browsed spruce. Letters denote significant differences among browse categories. Shaded regions reflect the 95% confidence interval around each regression line.

A total of 1,294 white spruce trees were measured (796 at Denali and 498 at Koyukuk) that varied in height (from 4 cm to > 800 cm) and age (from 5 to 135 years old). Widespread browsing of white spruce seedlings and saplings by hares occurred at both study locations; 40% (36% moderate browsing, 4% severe browsing) and 53% (34% moderate browsing, 19% severe browsing) of the measured white spruce had their apical meristem browsed at least once by hares at Denali and at Koyukuk, respectively. Most browsing events occurred when spruce were 10 to 20 years old for both Denali (mean age when browsed ± S.D., 15.8 ± 5.9 years) and Koyukuk (15.1 ± 8.1 years; Fig 2A). There was no significant difference in the age of browsed spruce between the study locations (paired t test, t = 0.07, d.f. = 51, *P* = 0.94).

A total of 988 spruce (76%) across both study regions were < 200 cm tall and this sample was used to analyze changes in height with changes in browsing history. Our model that included only the browsing by age interaction produced the lowest BIC score (Table 1). The interactive effect of spruce age and browsing history was significant (F = 45.34, d.f. = 2, 972.43, *P* < 0.001; Fig 2B). Moderately browsed (pairwise comparison of Least Squares Means, *P* <0.001) and severely browsed (*P* < 0.001) individuals had significantly different relationships between height and age than individuals that had not been browsed but did not differ from each other in their height-age relationship (*P* = 0.12). Both browse categories had reduced positive slopes for height with increasing spruce age than for individuals that were not browsed, but increases in the intercept for each browse category show that browsed young spruce (< 30 years of age) tended to be *taller* than unbrowsed individuals (Table 2). Our fixed effects explained 61% of the variation in spruce height ($R_{m}^{2}$ = 0.61). Removing browse history from the model reduced the amount of variation explained to 55% ($R_{m}^{2}$ = 0.55), suggesting browsing history explains a significant amount of variation in spruce height after accounting for spruce age. Plot and region explained an additional 18% of the variation in spruce height ($R_{c}^{2}$ = 0.78), suggesting that environmental conditions, not surprisingly, have a large effect on spruce growth.

**Table 1:** Six models were hypothesized to explain variation in spruce height at treeline (log-transformed). Final model selection was determined by calculating the Bayesian Information Criterion (BIC) for each model. Fixed effects included were spruce age (Age), browsing history (Browse), the proportion of associated deciduous woody vegetation browsed (pDeciduous), and habitat type (Habitat, upland or floodplain). Final model is indicated in bold.

Model	Fixed effects within model	BIC
Full	Age x Browse + pDeciduous + Habitat	1298.14
1	Age x Browse + pDeciduous	1292.41
2	Age x Browse + Habitat	1290.82
**3**	**Age x Browse**	**1285.83**
4	Age + Browse	1338.58
5	Age	1359.73

**Table 2:** Results of linear mixed effects model for white spruce seedling height at treeline in Alaska. The response variable, spruce height, was log-transformed.

Parameter	Estimate (± S.E.)	D.F.	t	*P*
*Intercept* (No browse)	2.020±0.089	2.50	22.72	<0.001
*Added intercept* (Moderate browse)	0.873±0.092	964.00	9.49	<0.001
*Added intercept* (Severe browse)	1.304±0.165	981.80	7.89	<0.001
*Slope* (Age x No browse)	0.085±0.002	980.30	31.14	<0.001
*Slope change* (Moderate browse)	-0.030±0.004	966.20	-7.71	<0.001
*Slope change* (Severe browse)	-0.044±0.005	966.20	-7.96	<0.001

## Discussion

Treeline white spruce trees are frequently browsed by snowshoe hares in Alaska. For newly recruiting individuals their fate is clear; a single browsing event can remove all meristematic tissue, resulting in death [4, 12]. For older seedlings with multiple growth points, browsing appears to have variable effects on spruce height, depending on the age of the individual. Although height growth occurs at a slower rate for browsed individuals as indicated by a reduced slope between spruce height and age, browsed spruce under approximately 30 years old tend to be *taller* than individuals that have not been browsed (Fig 2B). By contrast, after approximately 30 years of age, unbrowsed individuals surpass browsed individuals in height as a result of their faster height growth rate (greater slope), allowing them to reach 200 cm and escape hare browsing 5–10 years earlier than browsed spruce (Fig 2B).

The pattern of browsed spruce initially being taller than unbrowsed individuals could either reflect a response to herbivory or be the result of selective preferences of hares. Many deciduous species exhibit compensatory growth when browsed, and it is theoretically possible that spruce respond similarly to moderate levels of browsing [23]. Recent experiments using herbivore exclosures or clipping to simulate browsing, however, suggest white spruce does not exhibit a compensatory growth response [4, 24]. Browsed spruce may also benefit from the indirect effects of hare browsing on surrounding vegetation, but we found no support for the indirect effect of browsing on spruce height in our mixed effects model (Table 1).

Rather than being a response to herbivory, we surmise that greater initial height in browsed individuals reflects the browse preferences of hares. Hares are selecting to browse faster growing individuals that are taller [25, 26], whose faster growth may be a simple change in carbon allocation to biomass rather than defense (at a cost of greater vulnerability to herbivores). Because snow depth greatly influences the accessibility of spruce to browsing hares, faster growing seedlings that are taller will be exposed above the snowpack earlier than slower growing seedlings, increasing their likelihood of being browsed. In this study, 84% of browsed spruce were taller than the mean winter snow depth for Denali and the Koyukuk (~40cm, Western Regional Climate Center), whereas 55% of all unbrowsed spruce were below this height (Fig 2B). The expected advantages of growing faster at treeline are negated by increased vulnerability to browsing, which reduces spruce height, eventually reversing pre-existing height differences among browse categories (Fig 2B). Variable growth rates may also affect palatability and digestibility, where, as noted, trees allocating carbon to growth may produce less defensive compounds [24]. Conversely, slower growing spruce are likely to be more heavily defended chemically given reduced allocation of carbon to growth, and thus, avoided by hares. Suppression of faster-growing spruce will further slowdown treeline establishment in the presence of hares, as the time for populations of spruce to reach maturity and form a canopy (given sparse or less than spatially complete recruitment) will be determined by the slowest growing individuals in the population.

At both treeline locations, more browsing occurred in floodplain habitat than in upland habitat [12], suggesting that spruce growing in floodplain habitats are more likely to have reduced height because of browsing hares. This may partly explain why habitat type was not a significant variable in our final height model. Hare browsing may negate some of the expected increases in height for floodplain spruce. Exclosure studies have shown spruce growing under deciduous vegetation characteristic of floodplain habitats are shorter when exposed to hare herbivory than when excluded from herbivory [4]. At floodplain sites that are too open, spruce are known to be vulnerable to desiccation, resulting in reduced height growth at these locations [4, 27]. Thus, the potential for greater growth of spruce growing in productive floodplain habitats may be negated by hare browsing in areas with sufficient cover, and by physiological stress in open areas.

**Fig 3:**
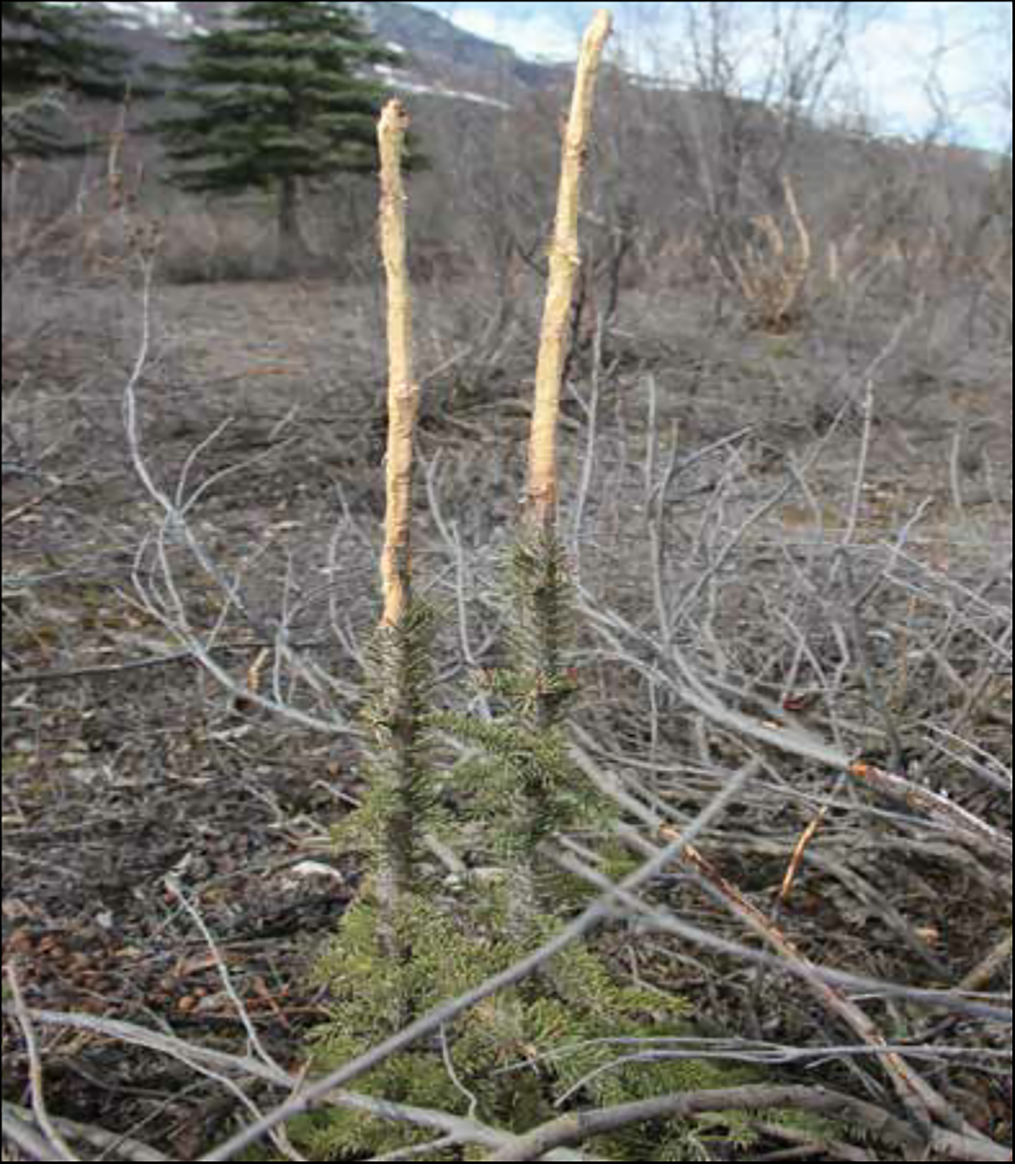
An unknown is the missing record of spruce that die because of hare browsing, as exemplified in the photograph of individuals likely to soon die by browsing hares at Koyukuk.

One component our study could not capture was the missing record of spruce seedlings that die because of hare browsing. At both locations we observed sites where seedlings had been killed due to browsing but remained standing (Fig 3). In the 7 plots (6 at Denali, 1 at Koyukuk) where we recorded spruce killed by hares, dead spruce made up to 53% (± 10%) of all standing spruce. This mortality estimate is conservative for these locations because many dead spruce, especially young seedlings, do not remain standing, as demonstrated in controlled experiments with planted spruce and herbivore exclosures [4]. The paucity of browsed spruce under ten years of age may partly reflect this missing record.

Our results also reinforce other findings that white spruce is an important component of the snowshoe hare winter diet [18, 19]. Whereas white spruce occurs at lower densities than most other potential woody forage plants, the proportion of individual spruce browsed (50%) was among the highest for any forage species (Table 3). Hares may actively select to browse spruce seedlings in the winter to help them to diversify their toxin load (albeit accruing additional toxins in the process). The importance of spruce as a winter diet item to hares should be emphasized, particularly in the context of the shifting growth performance of spruce across the Alaskan landscape in response to climate warming [28].

**Table 3:** The density of woody vegetation near treeline.

Species	Total Density, m^-2^	Browsed (%)
DENALI		
*Salix* sp.	3.93 ± 0.52*	47 ± 7%
*Betula nana*	1.66 ± 0.34	21 ± 3%
***Picea glauca***	**0.44 ± 0.05**	**40 ± 5%**
*Alnus* sp.	0.14 ± 0.07	30 ± 4%
*Shepherdia canadensis*	0.95 ± 0.25	10 ± 2%
*Populus balsamifera*	0.27 ± 0.08	35 ± 4%
*Rosa acicularis*	0.80 ± 0.22	15 ± 2%
KOYUKUK		
*Salix* sp.	3.63 ± 0.69	38 ± 5%
*Betula nana*	1.31 ± 0.49	23 ± 6%
***Picea glauca***	**0.59 ± 0.07**	**53 ± 6%**
*Alnus* sp.	0.70 ± 0.24	36 ± 7%
*Shepherdia canadensis*	1.45 ± 0.44	11 ± 3%
*Populus balsamifera*	0.27 ± 0.11	38 ± 7%
*Rosa acicularis*	0.04 ± 0.03	25 ± 3%

‘Total Density’ refers to the density of ramets or main stems for each species, including both browsed and unbrowsed individuals.

*Mean ± S.E

Understanding constraints on height growth is critical to understanding rates of treeline advance and the structure of the advancing forests. The effects of browsing by hares on spruce height have diverse functional consequences. Individual trees may appear to be initially benefitting from the presence of hares, either through compensatory growth following browse events or via indirect benefits of hares browsing surrounding vegetation. However, our results suggest that hares are selecting for taller, faster-growing individuals. Thus, the apparent positive effect may actually be an additional negative consequence, where the fastest-growing individuals in a population are suppressed by hares, causing the slower-growing individuals to determine the rate at which populations escape herbivory. Simultaneously, many young seedlings, as well as older individuals, succumb to severe hare browsing. Quantifying this missing record is key to understanding the capacity of herbivores to limit tree establishment, and for understanding the true potential of spruce to advance in the presence or absence of hares.

## Supporting information

S1 File Data files used in this study. This ZIP files contains all data used for this manuscript. Two files xml files are included, one for each study location.
(ZIP)
